# Comparison of the accuracy of residents, senior physicians and surrogate decision-makers for predicting hospital mortality of critically ill patients

**DOI:** 10.5935/0103-507X.20220019-en

**Published:** 2022

**Authors:** Bárbara Vieira Carneiro, Lucas Lonardoni Crozatti, Pedro Vitale Mendes, Antonio Paulo Nassar Júnior, Leandro Utino Taniguchi

**Affiliations:** 1 Emergency Medicine Discipline, Hospital das Clínicas, Faculdade de Medicina, Universidade de São Paulo - São Paulo (SP), Brazil.; 2 Intensive Care Unit, A.C. Camargo Cancer Center - São Paulo (SP), Brazil.

**Keywords:** Prognosis, Internship and residency, Critical illness, Decision-making, Delivery of health care, Hospitalization, Intensive care units, Mortality

## Abstract

**Objective:**

To compare the predictive performance of residents, senior intensive care unit physicians and surrogates early during intensive care unit stays and to evaluate whether different presentations of prognostic data (probability of survival versus probability of death) influenced their performance.

**Methods:**

We questioned surrogates and physicians in charge of critically ill patients during the first 48 hours of intensive care unit admission on the patient’s probability of hospital outcome. The question framing (i.e., probability of survival versus probability of death during hospitalization) was randomized. To evaluate the predictive performance, we compared the areas under the ROC curves (AUCs) for hospital outcome between surrogates and physicians’ categories. We also stratified the results according to randomized question framing.

**Results:**

We interviewed surrogates and physicians on the hospital outcomes of 118 patients. The predictive performance of surrogate decisionmakers was significantly lower than that of physicians (AUC of 0.63 for surrogates, 0.82 for residents, 0.80 for intensive care unit fellows and 0.81 for intensive care unit senior physicians). There was no increase in predictive performance related to physicians’ experience (i.e., senior physicians did not predict outcomes better than junior physicians). Surrogate decisionmakers worsened their prediction performance when they were asked about probability of death instead of probability of survival, but there was no difference for physicians.

**Conclusion:**

Different predictive performance was observed when comparing surrogate decision-makers and physicians, with no effect of experience on health care professionals’ prediction. Question framing affected the predictive performance of surrogates but not of physicians.

## INTRODUCTION

Prognostic assessment in critically ill patients is performed at intensive care unit (ICU) admission and daily thereafter for decisions concerning invasive treatments and for discussions with surrogate decision-makers (usually a family member). Although uncertainty and imprecisions are frequent in prognostication, most surrogates of patients understand this as unavoidable and still want a discussion of uncertain prognoses.^(1)^

However, this same uncertainty might hinder some physicians, especially those early in their career or in training, from engaging in further discussions. Therefore, a better understanding of physicians’ accuracy of prognostication could be informative.

Some previous studies suggested that ICU physicians were more accurate than severity-of-illness scores in predicting mortality.^(2)^ Surrogates might also be accurate but less accurate than physicians. This discordance about the patient’s prognosis might lead to and fuel conflicts.^(3)^ However, heterogeneity among these studies about the time of assessment (from less than 24 hours until 128 hours of ICU admission) and the outcome (from ICU mortality to 180-day mortality) makes this comparison problematic.^(2,3)^ The effect of previous experience and training on the discriminative performance of physicians has not been adequately evaluated. Finally, the format of prognostic data presentation might influence the surrogate’s perceived risks,^(4)^ but whether it influences the physician’s perceived risk is unknown.

Therefore, we conducted the present study to compare the prognostic ability of residents, senior ICU physicians, surrogates and severity of illness score early during ICU stay and to evaluate if different presentations of prognostic data (probability of survival *versus* probability of death) influences the prognostic ability of physicians’ categories and surrogates.

## METHODS

### Study design and patient population

This was a prospective cohort study of patients admitted to our medical-surgical ICU (a 14-bed unit at *Hospital das Clínicas*, a public tertiary universityaffiliated hospital located in São Paulo, Brazil) carried out from August 2017 to December 2019. The staffing model during the daytime included two senior ICU physicians, two ICU fellows and six internal medicine residents (who stayed for one month in the ICU during their internal medicine rotation). The inclusion criteria were admitted patients whose surrogate decision-makers could be interviewed ≤ 48 hours of ICU admission and one person ≥ 18 years old who identified as a surrogate decision-maker (not necessarily a first-degree family member). During the study period, each patient could be visited by two persons daily from 4 p.m. to 5 p.m., when physicians also approached them to discuss the patients’ clinical status. The exclusion criteria were patients younger than 18 years, pregnant patients and readmission in the same hospitalization.

### Data collection and study procedures

We enrolled patients whose surrogates had visited them at least once in the first 48 hours after ICU admission and had discussed the patient’s clinical status with the patients’ physician (usually the internal medicine resident and/or the ICU fellow). We interviewed participants only on weekdays (physicians could be interviewed from 4 p.m. to 5 p.m. and surrogates from 4 p.m. to 5 p.m. too, during the visit time). Research investigators approached the patients’ surrogates about study participation during the visitation period. Similar to a previous study, if more than one possible surrogate decision-maker was identified during the visitation, we interviewed the one who self-reported as having a significant amount of responsibility for decision-making.^(3)^ All participating surrogates and physicians provided written informed consent; for patients’ data, the ethical committee waived informed consent since all study information was collected from the administrative database already collected. The local Ethical Committee approved all study procedures (Ethics Committee approval 2.222.797, CAAE 69174717.4.0000.0068), and the study was performed according to national legislation (*Resolução* n° 466, December 12, 2012, Brazilian Ministry of Health).

Prior to the interview, we randomized the question framing (i.e., probability that the patient would survive the hospitalization or probability that the patient would die during the hospitalization) in a random sequence of two possibilities (i.e., simple randomization). The interview procedure consisted of asking the internal medicine resident, the ICU fellow and ICU senior physician in charge of the patient, as well as their surrogate decision-maker, to estimate the probability that the patient would survive/die the hospitalization. They were asked the validated question: “What do you think are the chances that the patient will survive/die this hospitalization?”^(3)^ They answered using a 0% to 100% scale in strata of 10% each (i.e., 0%, 10%, 20% and so on until 100%).^(3)^ All answers were blinded to the others’ responses. The interval for the research staff to obtain all answers did not exceed 1 hour.

We retrieved patients’ baseline characteristics and outcomes from our administrative database that was prospectively collected in a cloud-based software database (Sistema Epimed™) by trained staff.^(5)^ The data recorded included age, sex, date of ICU admission, Simplified Acute Physiology Score III (SAPS III),^(6,7)^ referring facility, admission diagnosis, surgical procedures before admission, Charlson index for comorbidities,^(8)^ Eastern Cooperative Oncology Group (ECOG), resource utilization during ICU stay (mechanical ventilation, vasoactive drugs or renal replacement therapy), oncological status (locoregional or metastatic) and hospital mortality. At the time of the interview, we also collected demographic data (age and sex) from the physicians and surrogate decision-makers. For the residents and ICU fellows, we also collected information about previous medical specialty, current residency and years since graduation. All participants’ senior ICU physicians were intensivists with complete training for more than five years and were certified by the National Board of Critical Care Medicine (*Associação de Medicina Intensiva Brasileira*).

### Sample size and statistical analysis

A previous systematic review suggested a discriminative performance estimated by a summary receiver operating characteristic (ROC) curve for physicians of 0.85 and 0.63 for severity-of-illness scores.^(2)^ We estimated that a sample size of 108 interviews would be required to detect a significant (i.e., significantly different from 0.5) discriminative performance of 0.63 (the lower value of the ROC curve in the previous systematic review), assuming a statistical power of 80%, an α = 0.05 and hospital mortality of 35% (hospital mortality of our ICU in the previous year).^(9)^ This sample size is also adequate to detect a significant difference between these predicted ROC values (0.85 *versus* 0.63) with the same hospital mortality, assuming a statistical power of 80% and α = 0.05 (in this case, 94 interviews would be required).^(9)^ To account for possible patients’ or surrogates’ consent withdrawal, we decided to enroll 10% more, for the final sample size estimation of 118 patients.

Continuous variables are presented as the median and interquartile range (IQR) and were evaluated using the Wilcoxon or Mann-Whitney tests. Categorical variables are presented as numbers and percentages and were evaluated using the chi-squared test.

To assess the accuracy of each group prediction, we obtained areas under the ROC curves (AUCs) for hospital outcome and compared them with the DeLong method without correction for multiple comparisons.^(10)^ Calibration was assessed by the calibration belt method as described by the *Gruppo Italiano per la Valutazione degli Interventi in Terapia Intensiva* (GiViTI). This method applies a generalized polynomial logistic function between the outcome and the logit transformation of the estimated predicted probability, with the respective 95 and 80% confidence intervals (CI) boundaries.

A statistically significant deviation from the bisector (the line of perfect calibration) occurs when the 95%CI boundaries of the calibration belt do not include the bisector.^(11)^ To calculate the calibration belt, 0% probability was converted to 0.01% and 100% probability was converted to 99.99%, since the method does not handle 0% and 100% probabilities from the questionnaire. A *post hoc* analysis was performed stratified by the use of invasive mechanical ventilation at the time of interview and comparing groups with SAPS III prediction using the standard equation for the probability of death.^(7)^ Statistical analyses were performed by using the software Statistical Package for the Social Sciences - SPSS (IBM Corp., Armonk, New York, USA), MedCalc for Windows, version 19.6 (MedCalc Software, Ostend, Belgium) and R (http://www.r-project.org).

## RESULTS

We enrolled 118 patients from August 2017 to December 2019 (Table 1). No consent withdrawals occurred. At enrollment, patients had a median age of 54 (IQR, 38.75 - 65.25), a median SAPS III score of 53 (IQR, 42.75 - 63), and 45 patients (38.1%) ultimately died in the hospital. At the time of the interview, a large proportion required invasive organ support (46.6% on mechanical ventilation and 50.8% on vasopressors), but no end-of-life discussion had taken place (Table 1). Two-thirds of the surrogates were women (74 of 118 surrogates) with a median age of 44.5 (IQR, 34 - 54). Most of them were spouses (31.4%) or siblings (35.6%, Table 2).

**Table 1:** Patient characteristics

Age (years)	54 (38.75 - 65.25)
Male	60 (50.8)
SAPS III	53 (42.75 - 63)
Admission type	
Medical	77 (65.3)
Emergency surgery	39 (33.1)
Elective surgery	2 (1.7)
Length of hospital stay before ICU admission (days)	1 (1 - 4)
Charlson comorbidity index	2 (0 - 3.5)
Eastern Cooperative Oncology Group	
0 - 1	85 (72.1)
2	23 (19.5)
3 - 4	7 (5.9)
Type of advanced disease	
Chronic heart failure (NYHA IV)	19 (16.1)
Cirrhosis	4 (3.4)
Metastatic cancer or hematologic malignancy	7 (5.9)
Sepsis at ICU admission^*^	29 (24.6)
Mechanical ventilation at interview†	55 (46.6)
Vasopressors at interview†	60 (50.8)
Renal replacement therapy at interview†	14 (11.9)
Decisions regarding limitation of treatment‡	
None	98 (83.1)
Any decision	12 (10.2)
Hospital mortality	45 (38.1)

SAPS III - Simplified Acute Physiology Score III; ICU - intensive care unit; NYHA - New York Heart Association.

# Sepsis was defined as suspected or confirmed infection with organ failure; † if the patient received that invasive organ support when the interview took place; ‡ data were missing for 8 patients. Results expressed as median (interquartile range) or n (%).

**Table 2:** Surrogate characteristics

Age (years)	44.5 (34 - 54)
Male	44 (37.6)
Relationship to patient	
Spouse	37 (31.4)
Child	42 (35.6)
Parent	14 (11.9)
Other relative	25 (21.2)
Level of education	
Did not attend school	4 (3.4)
Attended but did not complete school	38 (32.3)
Completed high school	45 (38.2)
Undergraduate degree	31 (26.3)
Religious preference	
Catholic	45 (38.1)
Evangelical	37 (31.4)
None, agnostic, or atheist	8 (6.8)
Spiritist	9 (7.6)
Other christian	2 (1.7)
Other	4 (3.4)
Declined response	4 (3.3)

Results expressed as median (interquartile range) or n (%).

We interviewed 18 residents, 8 ICU fellows and 5 senior ICU physicians. Residents who were interviewed had a median age of 26 (IQR, 25 - 27), and the majority were male (72.2%) with less than two years of graduation (84.7%). Intensive care unit fellow residents had a median age of 28 (IQR, 28 - 31), the majority were male (75%), 77.6% had less than four years of graduation and all had a previous residency in Internal Medicine. Intensive care unit senior physicians had a median age of 37 (IQR, 33.5 - 41.5), were mostly male (80.0%), and had a median time since graduation of 12 years (IQR, 10 - 19).

### Hospital outcome prediction

**Figure 1:**
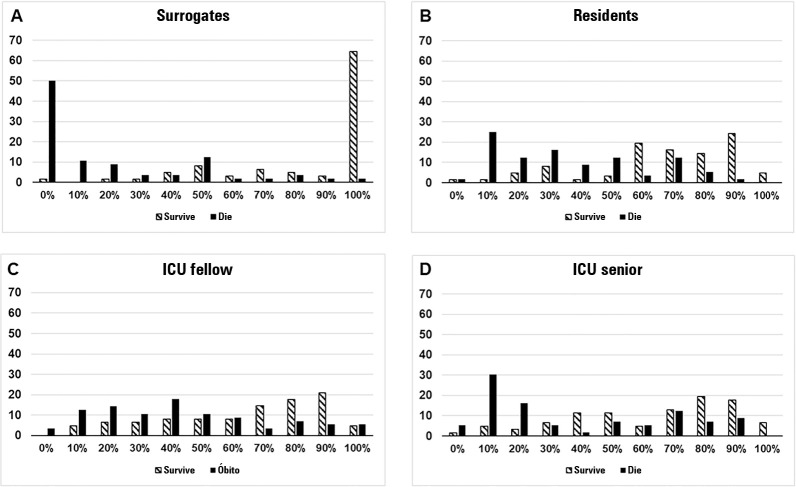
Estimated probabilities of hospital outcomes for patients by surrogates (A), residents (B), intensive care unit fellows (C) and intensive care unit senior physicians (D). ICU - intensive care unit.

We did not have any missing data about the predicted outcomes. The predicted probability of hospital outcomes differed between those who were interviewed (Figure 1 and Table 1S - Supplementary material).

Two correlated modes for the surrogates’ answers could be observed: a high percentage of “100% survival” and a high percentage of “0% dying”. For physicians, an even distribution in types of answers was observed.

**Figure 2:**
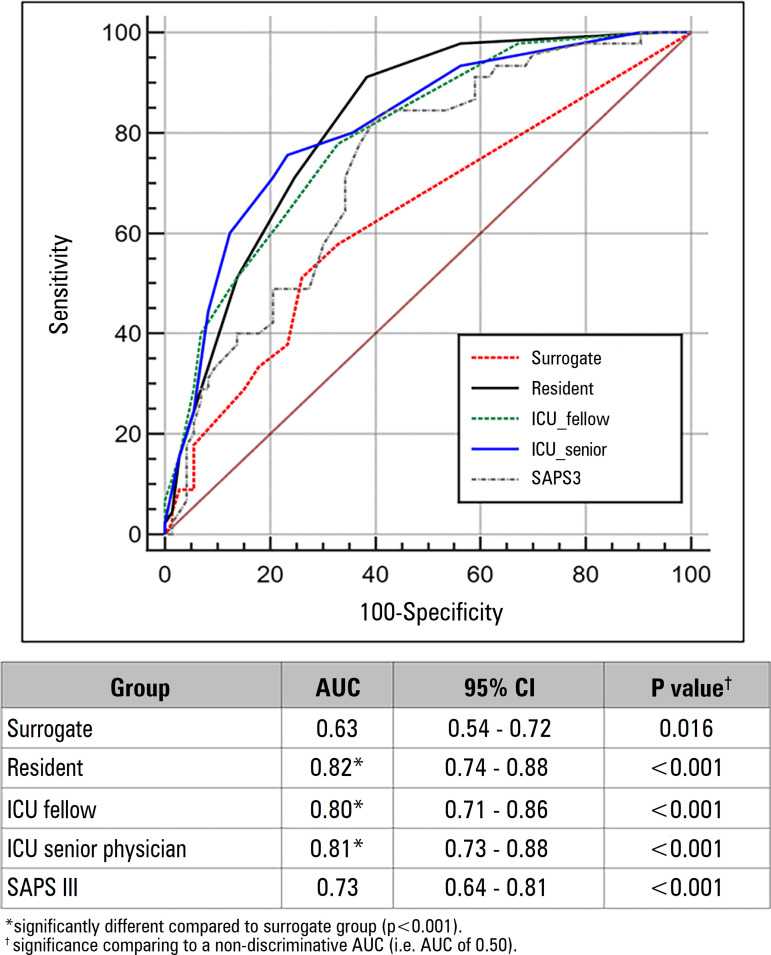
Discriminative performance for hospital outcomes by surrogates, residents, intensive care unit fellows, intensive care unit senior physicians and Simplified Acute Physiology Score III. ICU - intensive care unit; SAPS III - Simplified Acute Physiology Score III. AUC - area under the curve; 95%CI - 95% confidence interval.

Overall, all groups demonstrated a significant discriminative performance (i.e., different from 0.50), including SAPS III (Figure 2). Pairwise comparison showed a significantly higher discriminative performance between each physicians’ categories and surrogates’ (p < 0.001), but not between physicians’ categories themselves. In our *post hoc* analysis, the presence of invasive mechanical ventilation deteriorated the discriminative performance of physicians (Figure 1SA and 1SB - Supplementary material).

In all analyses, SAPS III performance was significantly different from 0.50 but was different from the surrogate prediction only in those without mechanical ventilation (Figure 1SB - Supplementary material). There was no difference between the SAPS III prediction and any of the physician groups.

Calibration analysis demonstrated that physicians’ estimates were calibrated (Figure 2S - Supplementary material). It also showed that surrogates’ estimates were miscalibrated over a wide range of predicted probabilities. SAPS III underestimated the mortality in the lower-mid range of predicted probabilities.

### Influence of question framing

All physicians’ categories’ predictive performance was different from chance regardless of the question framing. However, when surrogates were asked about the probability of dying, they were not accurate. ICU senior physicians demonstrated a higher AUC for both question framing compared to the surrogates (Table 3).

**Table 3:** Area under the receiver operating characteristics of interviewed groups to predict survival or death during hospitalization

	Surrogate	Resident		ICU fellow		ICU senior	
	Survive Die	Survive	Die	Survive	Die	Survive	Die
AUC	0.69^*^ 0.56	0.80^*^	0.82†^*^	0.80^*^	0.80†^*^	0.85‡^*^	0.79†^*^
95%CI	0.56 - 0.80 0.42 - 0.69	0.68 - 0.89	0.70 - 0.91	0.68 - 0.89	0.67 - 0.89	0.74 - 0.93	0.66 - 0.89

ICU - intensive care unit; AUC - area under the curve; 95%CI - 95%confidence interval.

* p < 0.001 for AUC significantly higher than 0.50; † p < 0.01 compared to surrogate’s prediction of dying in the hospitalization; ‡ p = 0.01 compared to surrogate’s prediction of surviving in the hospitalization.

## DISCUSSION

The main findings of our study are that (1) the predictive performance of both surrogate decision-makers and physicians for hospital mortality in our cohort of critically ill patients was significantly better than chance, but all physician groups (internal medicine residents, ICU fellows and ICU senior physicians) had better accuracy compared to surrogates, (2) physicians’ experience was not related to improvements in predictive performance, and (3) different question framing affected the surrogates’ predictive performance, notably if asked about the occurrence of death.

Sinuff et al. had previously performed a systematic review of studies that showed physicians’ predictive performance was better than that of severity-of-illness scores to predict outcomes after ICU admission for individual patients.^(2)^ Physicians’ observed predictive performance in our study was in the excellent range (0.80 - 0.90), as suggested by Hosmer et al.^(12)^ Although most studies are from the 1990s - 2000s, some recent observations also demonstrated physicians’ predictive performance similar to those in our study.^(3)^ Although different case mixes between studies could be observed [e.g., White et al. only studied patients on the fifth day of invasive mechanical ventilation,^(3)^ and the systematic review cited before enrolled studies with hospital mortality ranging from 18% to 46%^(2)^], it seems that physicians’ estimates of prognosis are accurate.

Although we could not demonstrate a statistically significant difference between SAPS III performance and the physicians’ performance as previously described,^(2)^ this might be due to a lack of power in our *post hoc* analysis. Nevertheless, in all conditions, the point estimate of physicians’ performance was higher than that observed for SAPS III.

We did not observe significant differences between senior physicians and junior residents. Previous publications did not demonstrate that experienced ICU physicians demonstrate better predictive performance than inexperienced physicians.^(2)^ Gusmão Vicente et al. did not observe a statistically significant difference between senior and junior ICU physicians in predicting longer ICU stays and ICU outcomes.^(13)^ Even when comparing only trainees, Kruse et al. observed the same ROC curve for fellows, residents and interns.^(14)^ Our results are similar since experienced (senior physicians) and inexperienced (fellows and residents) physicians had similar predictive performances and they were all well calibrated.

We also observed that the overall predictive performance of surrogates was lower than those observed from physicians in our study. Notably, the calibration was poor in a wide range of predicted estimates, as observed in the calibration belt. This is noteworthy and highly anticipated, since it is not expected that a nonmedically educated person (who also has feelings involved for the person they are representing) can be compared to health care professionals. However, this fact is clinically relevant, as White et al. demonstrated that this divergent predictive performance is associated with discordances between prognostic points of view.^(3)^ Family members are usually overoptimistic due to both misunderstanding of the prognostic information conveyed by physicians and optimistic biases,^(3,15)^ and these factors might lead to conflict and the overuse of inappropriate invasive support. Thus, it is highly suggested to start any serious discussion by asking the patient and family members’ point of view and their actual perceptions.^(16)^ Our data emphasize this possible source of discordance, since we also observed that surrogates usually conveyed optimistic answers, with a high number of “100% chance of surviving” and “0% chance of dying” answers.

An interesting finding of our study was the effect of question framing on the predictive performance among surrogates but not on physicians. It was formerly demonstrated that a prognostic data presentation format might influence how surrogate decision-makers perceive risk, especially when the data are qualitatively explained,^(4)^ but this effect was not previously studied for physicians.

Most publications presented the question in a survival frame (i.e., the probability that the patient will survive the hospitalization).^(14,17,18)^ However, the effect of question framing was not previously studied. It is well known that risk perception is highly variable among subjects due to multiple factors.^(19)^ In the psychometric paradigm, the individual quantitatively judges the current and desired riskiness.^(20)^ However, in situations where no a priori experience exists (such as ICU admission), personal beliefs and feelings are likely to be influenced by the framing of presentations.^(20)^ We believe that this should be taken into account when presenting prognostic information to surrogates and should also be acknowledged when designing future qualitative studies.

Another unexpected finding was the deterioration in the physicians’ category accuracy when faced with patients under mechanical ventilation. The reasons for the deterioration are unknown. Although a type I error cannot be excluded in this post hoc analysis, previous studies demonstrated inaccuracy in prognostication for some aspects of mechanically ventilated patients. Figueroa-Casas et al. demonstrated that physicians’ accuracy in predicting the duration of mechanical ventilation was limited.^(21)^ Young et al. also suggested that the ability of physicians to predict extended ventilation and the requirement for a tracheostomy was poor.^(22)^ Therefore, additional care might be taken when predicting outcomes for patients on mechanical ventilation, since some judgment bias could be present.

Our study has some limitations. First, it is a singlecenter study performed in a university-affiliated public hospital. Therefore, our results are influenced by our local case mix of patients, physicians and surrogates. This should be taken into account when comparing our data with others. Second, we did not interview health care professionals other than physicians to predict survival. A previous publication suggested that the perception of nurses is also relevant.^(23)^ Third, the lack of a predictive performance difference between senior physicians and residents might be due to a lack of power to detect smaller differences than the one observed. However, previous publications documented similar findings.^(13,14)^ Finally, we presented the prognostic question as strata of numerical risk. Numeracy is critical to proper understanding of such a presentation format,^(24,25)^ and it is particularly relevant in low- and middle-income countries where low educational attainments are unfortunately frequent.

## CONCLUSION

In conclusion, we observed higher predictive performance for hospital outcomes among physicians than among surrogate decision-makers, who are usually overly optimistic. Experience did not lead to better predictive performance among the included physicians. On the other hand, question framing (i.e., probability of death *versus* probability of survival) influenced surrogate performance.
