# Supplementary material for: Comparison of the accuracy of residents, senior physicians and surrogate decision-makers for predicting hospital mortality of critically ill patients
Source: Rev Bras Ter Intensiva. 2022 Apr-Jun;34(2):220–6. doi: 10.5935/0103-507X.20220019-en (PMC9354110; doi:10.5935/0103-507X.20220019-en)
Supplement: Supplementary file 1 [file rbti-34-02-0220-suppl01.pdf]

Comparison of the accuracy of residents, senior physicians and surrogate decision-makers for predicting hospital mortality of critically ill patients

Comparação da acurácia de residentes, médicos seniores e decisores substitutos na previsão da mortalidade hospitalar de pacientes críticos

Bárbara Vieira Carneiro[1], Lucas Lonardoni Crozatti[1], Pedro Vitale Mendes[1], Antonio Paulo Nassar Júnior[2], Leandro Utino Taniguchi[1]

Table 1S - Estimated probabilities of survival or death by interviewed groups

Estimate %	Prediction by surrogate n (%)		Prediction by resident n (%)		Prediction by ICU fellow n (%)		Prediction by ICU senior n (%)	
	Survive	Die	Survive	Die	Survive	Die	Survive	Die
0	1 (1.6)	28 (50)	1 (1.6)	1 (1.8)	--	2 (3.6)	1 (1.6)	3 (5.4)
10	--	6 (10.7)	1 (1.6)	14 (25)	3 (4.8)	7 (12.5)	3 (4.8)	17 (30.4)
20	1 (1.6)	5 (8.9)	3 (4.8)	7 (12.5)	4 (6.5)	8 (14.3)	2 (3.2)	9 (16.1)
30	1 (1.6)	2 (3.6)	5 (8.1)	9 (16.1)	4 (6.5)	6 (10.7)	4 (6.5)	3 (5.4)
40	3 (4.8)	2 (3.6)	1 (1.6)	5 (8.9)	5 (8.1)	10 (17.9)	7 (11.3)	1 (1.8)
50	5 (8.1)	7 (12.5)	2 (3.2)	7 (12.5)	5 (8.1)	6 (10.7)	7 (11.3)	4 (7.1)
60	2 (3.2)	1 (1.8)	12 (19.4)	2 (3.6)	5 (8.1)	5 (8.9)	3 (4.8)	3 (5.4)
70	4 (6.5)	1 (1.8)	10 (16.1)	7 (12.5)	9 (14.5)	2 (3.6)	8 (12.9)	7 (12.5)
80	3 (4.8)	2 (3.6)	9 (14.5)	3 (5.4)	11 (17.7)	4 (7.1)	12 (19.4)	4 (7.1)
90	2 (3.2)	1 (1.8)	15 (24.2)	1 (1.8)	13 (21.0)	3 (5.4)	11 (17.7)	5 (8.9)
100	40 (64.5)	1 (1.8)	3 (4.8)	--	3 (4.8)	3 (5.4)	4 (6.5)	--

ICU - intensive care unit.

A **With mechanical ventilation**

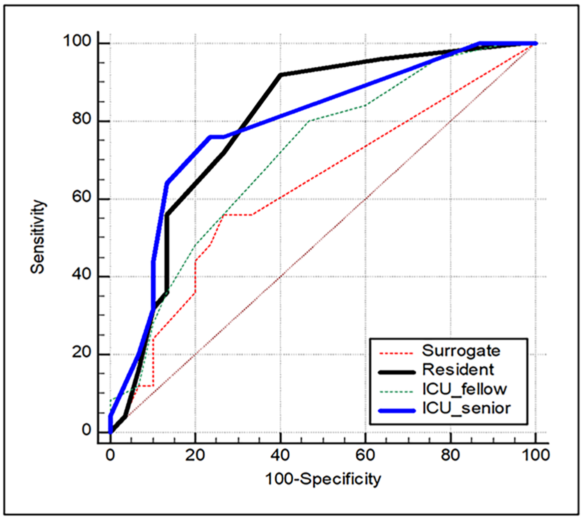

Group	AUC	95% CI	P value†
**Surrogate**	0.63	0.49 - 0.75	0.07
**Resident**	0.80*	0.67 - 0.89	<0.001
**ICU fellow**	0.71	0.57 - 0.83	0.002
**ICU senior physician**	0.79#	0.66 - 0.89	<0.001

*significantly different compared to surrogate group (p=0.026).
#significantly different compared to surrogate group (p=0.015)
†significance comparing to a non-discriminative AUC (i.e. AUC of 0.50).

B **No mechanical ventilation**

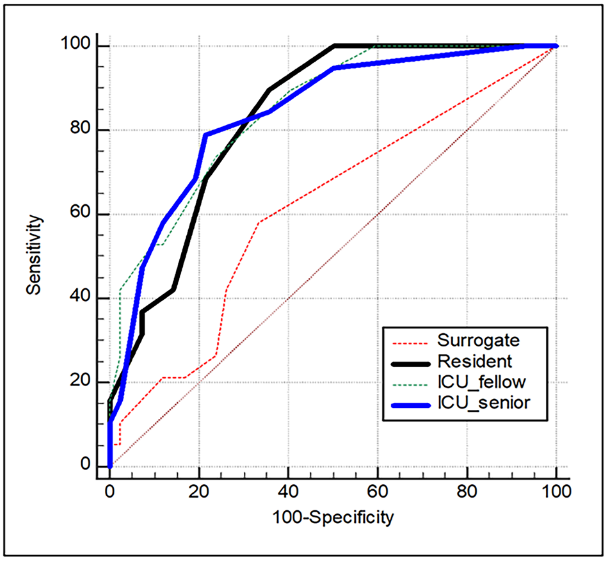

Group	AUC	95% CI	p value†
**Surrogate**	0.61	0.48 - 0.74	0.11
**Resident**	0.83*	0.72 - 0.92	<0.001
**ICU fellow**	0.85*	0.73 - 0.93	<0.001
**ICU senior physician**	0.84*	0.72 - 0.92	<0.001

*significantly different compared to surrogate group (p<0.01).
†significance comparing to a non-discriminative AUC (i.e. AUC of 0.50).

Figure 1S - Discriminative performance for hospital outcomes by surrogates, residents, intensive care unit fellows and intensive care unit senior physicians, stratified by the presence (A) or not (B) of invasive mechanical ventilation.

ICU - intensive care unit; AUC - area under the curve; 95%CI - 95% confidence interval.

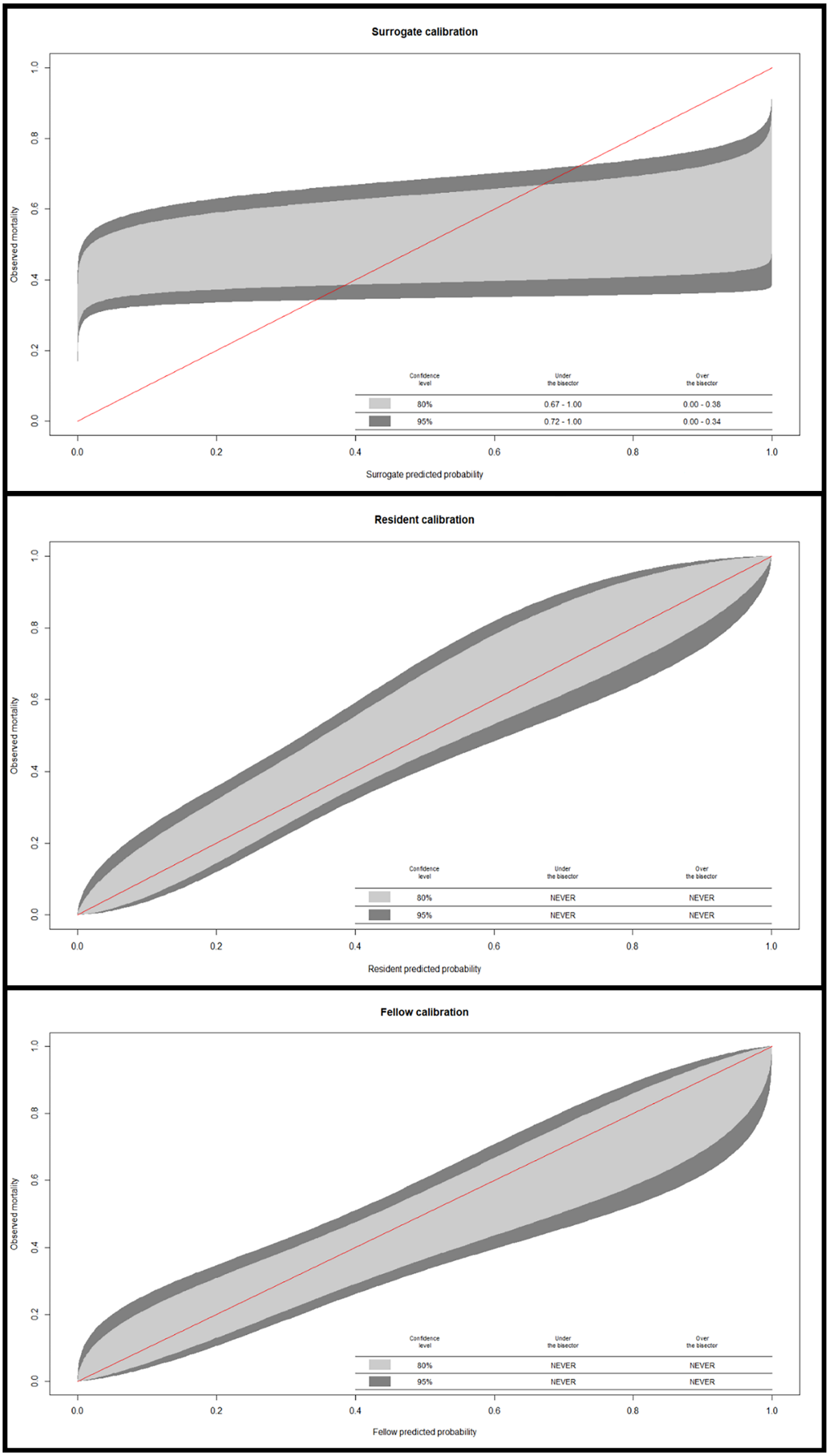
Surrogate calibration
Observed mortality
0.0
0.2
0.4
0.6
0.8
1.0
Confidence level
Under the bisector
Over the bisector
80%
0.67 - 1.00
0.00 - 0.38
95%
0.72 - 1.00
0.00 - 0.34
Surrogate predicted probability
Resident calibration
Observed mortality
Confidence level
Under the bisector
Over the bisector
80%
NEVER
NEVER
95%
NEVER
NEVER
Resident predicted probability
Fellow calibration
Observed mortality
Confidence level
Under the bisector
Over the bisector
80%
NEVER
NEVER
95%
NEVER
NEVER
Fellow predicted probability

Continue...

...continuation

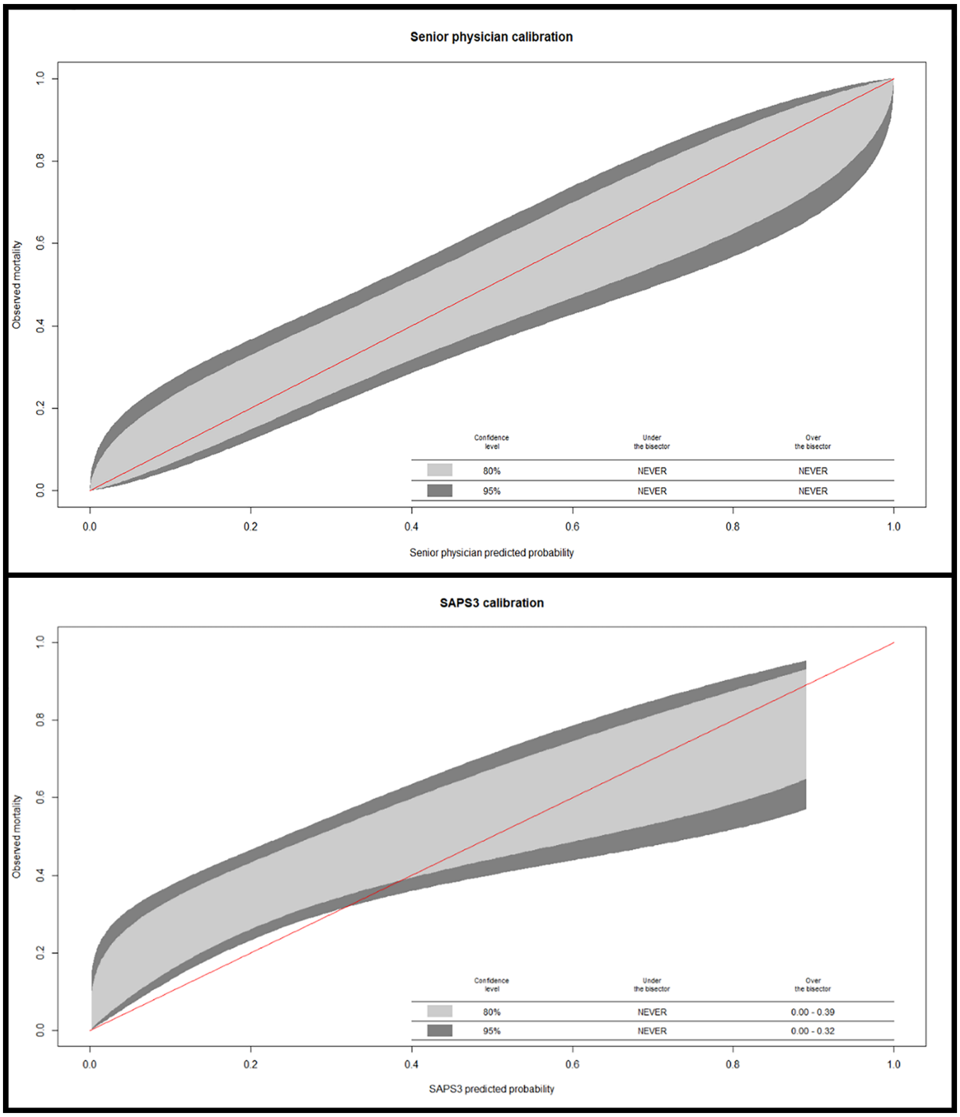

Figure 2S - Calibration belt for surrogates, residents, intensive care fellows, senior physicians and Simplified Acute Physiology Score III.

SAPS 3 - Simplified Acute Physiology Score III.
